# Sex-based analysis of treatment responses in animal models of sepsis: a preclinical systematic review protocol

**DOI:** 10.1186/s13643-023-02189-2

**Published:** 2023-03-21

**Authors:** MengQi Zhang, Dean A. Fergusson, Rahul Sharma, Ciel Khoo, Asher A. Mendelson, Braedon McDonald, Kimberly F. Macala, Neha Sharma, Sean E. Gill, Kirsten M. Fiest, Christian Lehmann, Risa Shorr, Forough Jahandideh, Stephane L. Bourque, Patricia C. Liaw, Alison Fox-Robichaud, Manoj M. Lalu, on behalf of the Canadian Critical Care Translational Biology Group, the Sepsis Canada National Preclinical Sepsis Platform, Marc T. Avey, Emmanuel Charbonney, Arnold Kristof, Gloria Vazquez-Grande, Ruud Veldhuizen, Brent Winston, Salman Qureshi, Juan Zhou

**Affiliations:** 1 Faculty of Medicine, University of Ottawa, 451 Smyth Rd #2044, Ottawa, ON K1H 8M5 Canada; 2 Clinical Epidemiology Program, Blueprint Translational Group, Ottawa Hospital Research Institute, 501 Smyth Box 511, Ottawa, ON K1H 8L6 Canada; 3 Department of Internal Medicine, Section of Critical Care Medicine, Rady Faculty of Health Sciences, University of Manitoba, 820 Sherbrook Street, Winnipeg, MB R3A 1R9 Canada; 4 Department of Critical Care Medicine, Cumming School of Medicine, University of Calgary, 3330 Hospital Drive NW, Calgary, AB T2N 4N1 Canada; 5 Snyder Institute for Chronic Diseases, Cumming School of Medicine, University of Calgary, 3280 Hospital Drive NW, Calgary, AB T2N 4N1 Canada; 6 Department of Critical Care Medicine, Royal Alexandra Hospital, University of Alberta, 2-214 Clinical Science Building, 8440-112Th Street, Edmonton, AB T6G 2B7 Canada; 7 Department of Medical Sciences and Thrombosis and Atherosclerosis Research Institute, McMaster University, 237 Barton St East, Hamilton, ON L8L 2X2 Canada; 8 Centre for Critical Illness Research, Lawson Health Research Institutes, Victoria Research Labs, A6-134, 800 Commissioners Road Ease, London, ON N6A 5W9 Canada; 9 Division of Respirology, Department of Medicine, Western University, London, ON Canada; 10 Department of Anesthesia, Pain Management and Perioperative Medicine, II Health Sciences Centre, 5850 College Street, Halifax, NS B3H 1X5 Canada; 11 Learning Services, The Ottawa Hospital, Ottawa, ON Canada; 12 Department of Anesthesiology & Pain Medicine, Katz Group Centre for Pharmacy and Health Research, University of Alberta, 3-020H, Edmonton, AB T6G 2E1 Canada; 13 Department of Medicine and Thrombosis and Atherosclerosis Research Institute, McMaster University and Hamilton Health Sciences, 237 Barton St East, Hamilton, ON L8L 2X2 Canada; 14 Department of Anesthesiology and Pain Medicine, The Ottawa Hospital, Room B307, 1053 Carling Avenue, Mail Stop 249, Ottawa, ON K1Y 4E9 Canada; 15 Regenerative Medicine Program, Ottawa Hospital Research Institute, 501 Smyth Box 511, Ottawa, ON K1H 8L6 Canada

**Keywords:** Sex, Sepsis, Intervention, Animal models, Systematic review

## Abstract

**Background:**

The importance of investigating sex- and gender-dependent differences has been recently emphasized by major funding agencies. Notably, the influence of biological sex on clinical outcomes in sepsis is unclear, and observational studies suffer from the effect of confounding factors. The controlled experimental environment afforded by preclinical studies allows for clarification and mechanistic evaluation of sex-dependent differences. We propose a systematic review to assess the impact of biological sex on baseline responses to disease induction as well as treatment responses in animal models of sepsis. Given the lack of guidance surrounding sex-based analyses in preclinical systematic reviews, careful consideration of various factors is needed to understand how best to conduct analyses and communicate findings.

**Methods:**

MEDLINE and Embase will be searched (2011-present) to identify preclinical studies of sepsis in which any intervention was administered and sex-stratified data reported. The primary outcome will be mortality. Secondary outcomes will include organ dysfunction, bacterial load, and IL-6 levels. Study selection will be conducted independently and in duplicate by two reviewers. Data extraction will be conducted by one reviewer and audited by a second independent reviewer. Data extracted from included studies will be pooled, and meta-analysis will be conducted using random effects modeling. Primary analyses will be stratified by animal age and will assess the impact of sex at the following time points: pre-intervention, in response to treatment, and post-intervention. Risk of bias will be assessed using the SYRCLE’s risk-of-bias tool. Illustrative examples of potential methods to analyze sex-based differences are provided in this protocol.

**Discussion:**

Our systematic review will summarize the current state of knowledge on sex-dependent differences in sepsis. This will identify current knowledge gaps that future studies can address. Finally, this review will provide a framework for sex-based analysis in future preclinical systematic reviews.

**Systematic review registration:**

PROSPERO CRD42022367726.

**Supplementary Information:**

The online version contains supplementary material available at 10.1186/s13643-023-02189-2.

## Introduction

Sepsis is the life-threatening systemic host response to infection and is estimated to contribute to 1 in 5 annual deaths worldwide [1]. Appropriately, the World Health Organization has recognized sepsis as a global health priority [2]. Hospitalized patients who acquire sepsis have significantly increased death, hospital readmission, and healthcare costs [3]. Despite decades of research, mortality rates for extreme forms of sepsis have remained relatively stagnant at approximately 25–30% [2]. Notably, there are no experimental therapies currently approved for the treatment of sepsis, despite numerous candidates demonstrating promise in preclinical studies [4, 5].

One unresolved issue in sepsis is the influence of biological sex on pathophysiology and outcomes (e.g., mortality). This is particularly important as sex bias may contribute to the failures of translational sepsis research [4, 6]; clinical evidence investigating sex-dependent differences in sepsis is equivocal, and two systematic reviews on this topic encouraged further investigation [7, 8]. These clinical studies are also limited as they are restricted to observational data that are subject to confounding factors (e.g., comorbidities of patients).

Preclinical (i.e., laboratory-based) studies provide an opportunity to overcome and address key limitations of available clinical evidence on this topic. The laboratory setting allows for precise control of potential confounders to better understand associations between variables such as biological sex and biochemical, pathologic, and physiologic outcomes. Preclinical research to date has suggested that female animals may be protected in sepsis compared to males, with many potential mechanisms underpinning these differences (e.g., X-linked mosaicism, protective effects of estrogen) [9]. Our previous systematic review investigating sex-dependent differences in response to the cornerstones of sepsis therapy (antibiotics and fluids) revealed a paucity of studies on this topic [6]. As such, our proposed systematic review expands on this work by investigating sex differences in sepsis outcomes after induction of disease, both prior to and in response to all interventions. Specifically, our primary question is “What is the effect of biological sex on inflammation, organ dysfunction, and mortality in in vivo animal models of sepsis?” Given the lack of guidance on sex-based analysis in preclinical systematic reviews, we will also explore and compare various approaches to report results; illustrative examples are provided in this protocol. Our systematic review will provide a foundational understanding of sex-dependent differences in preclinical sepsis and will inform future preclinical and clinical studies that seek to evaluate novel interventions.

## Methods and design

### Review team

We have assembled an interdisciplinary team for this systematic review. Members have expertise in systematic reviews (D. A. F., M. M. L., J. M.), preclinical (M. M. L., A. A. M., B. M., C. L., N. S., S. E. G., P. C. L., A. F. R., F. J., S. L. B), and clinical research (K. M. F., M. M. L., A. A. M., A. F. R., K. F. M.). Students for this project (M. Z., R. S., C. K.) have experience in basic science and are receiving training in clinical research.

### Protocol

Our protocol is registered on PROSPERO, the International Prospective Register of Systematic Reviews (https://www.crd.york.ac.uk/PROSPERO/; registration number CRD42022367726). This protocol manuscript was prepared in accordance with the PRISMA-P checklist [10] (see Additional file 1: Appendix 1 for full checklist). Any post-protocol adjustments will be reported in the final manuscript.

### Terminology

Given the prevalence of incorrect usage of terminology in sex- and gender-based research, we have listed definitions for several key terms, adapted from the Canadian Institutes of Health Research [11]:

1. *Sex* refers to a set of biological attributes in humans and animals. It is primarily associated with genetically regulated physical and physiological features including chromosomes, gene expression, hormone levels and function, and reproductive/sexual anatomy. Sex is usually categorized as female or male, but there is variation in how these attributes are expressed.
2. *Gender* refers to the socially constructed roles, behaviors, expressions, and identities of girls, women, boys, men, and gender-diverse people. It influences how people perceive themselves and each other, how they act and interact, and the distribution of power and resources in society. Gender is often conceptualized as binary in research (e.g., girl/woman and boy/man), but there is variation.
3. *Sex- and gender-based analysis* is an approach that systematically examines sex-based (biological) and gender-based (sociocultural) differences between men, women, boys, girls, and gender-diverse people.

In preclinical research, “sex” is almost always the correct term to be used in the study of nonhuman animals [12]. The incorrect use of these terms is widespread, which is one contributing factor to these variables remaining relatively unexplored and misunderstood [13, 14].

### Eligibility criteria

#### Animals and models

- Inclusion—In vivo animal models of experimentally induced, acute sepsis. Only models representing true infectious sepsis will be included (e.g., cecal ligation and puncture, fecal-induced peritonitis). Animals of all ages will be included. All mammalian species will be included (e.g., mice, rats, pigs, sheep). However, animals will be classified by age (e.g., neonatal, adult, or aged mice), and separate primary analyses will be conducted for each of these groups.
- Exclusion—In vitro, ex vivo, and nonmammalian/invertebrate animal models will be excluded. Noninfectious models of sepsis will also be excluded (e.g., lipopolysaccharide administration) as current recommendations suggest only true infections should be used to model sepsis [15].

#### Exposure

- Inclusion—Studies reporting data stratified by male and female sex
- Exclusion—Studies that do not report data stratified by sex

#### Intervention

- Inclusion—Any intervention that is hypothesized to improve sepsis-induced outcomes will be included. These will include pharmacological interventions (e.g., drugs, supplements, hormones, steroids, antibodies), non-pharmacological interventions (e.g., mesenchymal stromal cell therapy, extracellular vesicle therapy), and other interventions (e.g., gene manipulation such as knockout/knock-in animal models). Assessing all interventions in our systematic review will allow for a broad understanding of sex-dependent factors that may exist in sepsis. With that, we appreciate that the diversity of underlying mechanisms that may contribute to potential sex-dependent differences complicates the interpretation of these analyses. As a result, we will conduct subgroup analyses to delineate any dimorphic responses to treatment between males and females specific to the category of intervention. Interventions may be administered at any timepoint, including pre-treatment, at time of sepsis induction, or as rescue. Finally, we will also consider studies that reported baseline data in male and female animals following the establishment of sepsis.
- Exclusion—Any interventions hypothesized or demonstrated to contribute to the pathogenesis of sepsis or worsen outcomes of sepsis will be excluded.

#### Comparators

- Inclusion—Studies with any comparator or no comparator will be included, provided that sex-stratified data is reported; for example, studies that only report sex-stratified baseline data (i.e., sepsis only, with no intervention of interest) or sex-stratified data for groups that all receive the intervention of interest.
- Exclusion—Comparisons to non-septic animals

#### Outcomes

Inclusion—Studies that report sex-stratified data for any of the following outcomes will be included as follows:

1. Death (or humane surrogate endpoints) will be the primary outcome. Given the variety of methods that death is reported, we will consider death at the latest timepoint reported and by pre-specified time intervals: less than 2 days, between 2 and 4 days, and greater than 4 days. We have examined mortality at these pre-specified time points in previous preclinical systematic reviews of sepsis and critical illness [16, 17]. This will provide a deeper understanding of potential differences in the progression of disease between males and females.
2. Organ dysfunction and injury (secondary outcome) will be organized by system; for example, pulmonary dysfunction (e.g., histological evidence of tissue injury; alteration of the alveolar-capillary barrier as measured by total protein, albumin, and IgM levels in bronchoalveolar fluid), renal dysfunction (e.g., creatinine, urea), hepatic dysfunction (e.g., AST, ALT), and cardiac dysfunction (echocardiographic assessment of function, troponin I or T). When possible, standardized and/or validated scoring systems of organ injury will be noted.
3. Bacterial load (secondary outcome) will be assessed by colony-forming units in specific organs or sites (e.g., peritoneal fluid, blood, spleen).
4. IL-6 (secondary outcome) will be reported as a measure of overall inflammation. Organ dysfunction and IL-6 will be described at less than 6 h, between 6 and 24 h, and later than 24 h. These windows of ascertainment have been used in our previous systematic reviews of animal models of critical illness [16, 17].

Studies which investigate either ovariectomized females or castrated males will be included in our review but will not be pooled in primary analyses.

#### Study design, publication type

- Inclusion—In vivo, interventional studies with or without a comparator or control will be included. Studies may be randomized, non-randomized, or pseudo-randomized. Only published journal articles of primary research will be included.
- Exclusion—Abstracts, review articles, letters, editorials, unpublished gray literature, and studies involving solely in vitro or ex vivo data will be excluded.

### Data sources

Ovid MEDLINE and Embase will be searched for the past 10 years. Current recommendations for clinical systematic reviews suggest MEDLINE, Embase, and CENTRAL should be searched [18]; however, since CENTRAL does not index preclinical/animal studies, it will not be included. The date restriction was implemented for two primary reasons. Firstly, we anticipate that the majority of relevant studies will be published in this period, since major funding agencies have only recently emphasized the importance of investigating sex-dependent differences. Secondly, feasibility and resource limitations prevent us from conducting a search from inception. Reference lists of included studies and relevant reviews will be manually reviewed.

### Search strategy

Search strategies (Additional file 1: Appendix 2) will be designed and developed with the assistance of an information specialist with expertise in development of preclinical systematic search strategies (Risa Shorr, MLS, Ottawa Hospital Library Learning Services). In the development of the search, key words related to in vivo preclinical studies including calculated vocabulary (e.g., sepsis) and acronyms (e.g., CLP) will be used. Additional modifications will be added to the search on a per database basis. Filters validated for preclinical animal studies will be applied to improve search efficiency. We will search MEDLINE (OVID interface, including In-Process and Epub Ahead of Print) and Embase (OVID interface). Only articles published in English will be included in this review. A sample search strategy is provided in Additional file 1: Appendix 2.

### Study records

#### Data management

Studies captured by our search will be uploaded to DistillerSR (Evidence Partners, Ottawa, Canada), which is a cloud-based and audit-ready platform.

#### Study selection

Titles and abstracts will be screened independently and in duplicate using the inclusion criteria described. A calibration test utilizing 50 studies will be performed to refine the screening criteria and ensure high inter-rater correlation, prior to commencement of screening. Subsequently, full-text articles will be retrieved for articles that meet inclusion criteria or where there is any uncertainty. Prior to commencement of full-text screening, a second calibration test utilizing a set of 10 full-text articles will be conducted. Full-text articles will be screened for inclusion independently and in duplicate. Any inclusion conflicts identified by either of the two calibration tests will be resolved through consultation with the entire review team. Following refinement of full-text screening criteria, formal screening will commence. To resolve any inclusion conflicts between reviewers, a senior author will be consulted. Relevant justification will be recorded for exclusion of any full-text articles.

#### Data collection process and data items

Standardized data extraction forms will be created using DistillerSR. Relevant data items will be extracted independently by one reviewer and audited by a second independent reviewer. Categories of data items to be collected are outlined in Table 1. Prior to formal extraction, a calibration exercise using three studies will be performed to refine the data extraction form. To extract and quantify data presented only in graphical format, Engauge Digitizer will be used. Any conflicts will be resolved through consensus between the reviewer and auditor, and if necessary, a third party will be consulted if no resolution can be made. In instances with uncertainty, study authors will be contacted for clarification.

**Table 1:** Data collection elements

Category of interest	Example items
Study characteristics	Methods, setting, sample size, number of experimental groups, number of animals per group
Publication characteristics	Study title, first author, date of publication, journal, funding support, country, number of total authors
Study population (animal model)	Age, weight, species (e.g., mouse, rat, pig, dog), strain, number of animals
Type of sepsis model	Cecal ligation and puncture, fecal slurry injection, live bacterial injection, colon ascendens stent peritonitis
Intervention and comparison characteristics	Type of intervention, dose, timing, route of administration
Co-interventions	Antibiotics, fluid resuscitation, vasopressors
Outcomes	Death (or humane surrogate endpoints, measured in number of deaths (dichotomous)). Death will also be described at less than 2 days, between 2 and 4 days, and greater than 4 days to better understand the natural course of disease between male and female animal models Organ dysfunction: pulmonary (histological evidence of tissue injury, measured using histological scores), alteration of the alveolar capillary barrier, measured via total protein, albumin, and IgM, renal (creatinine or urea), hepatic (AST, ALT), cardiac (echocardiographic assessment of left ventricular ejection fraction, fractional shortening, troponin I or T) Bacterial load — colony-forming units per milliliter in sample organs/sites (e.g., peritoneal fluid, spleen) IL-6 levels
Risk-of-bias items	In accordance with a modified version of SYRCLE’s risk-of-bias tool: selection bias, performance bias, detection bias, attrition bias, reporting bias
Other	Type of comparator

### Risk-of-bias assessment

To assess risk of bias, we will use a modified version of the Systematic Review Centre for Laboratory Animal Experimentation (SYRCLE)’s risk-of-bias tool [19]. This tool was specifically designed and validated for assessing risk of bias in preclinical animal studies. Items include concealment of allocation, random sequence generation, description of baseline characteristics of animals, blinding of personnel and endpoint measurements, selective outcome reporting, and completeness of endpoint reporting. Members of our team have used this modified version and found it feasible [20, 21]. Each domain for all studies will be assessed and assigned a value of low, high, or unclear risk of bias. Risk of bias will be assessed independently and in duplicate by two reviewers. Any conflicts will be resolved through discussion with a senior author.

### Data analysis

When appropriate, categorical variables will be summarized by frequencies/percentages, and continuous variables will be summarized by means and standard deviations or median and interquartile ranges depending on the distribution of data. We will qualitatively assess studies initially for heterogeneity in models of sepsis used, methods used to ascertain outcomes, etc. and potentially restrict our analysis to synthesis without meta-analysis. Studies included in meta-analysis will be pooled using Comprehensive Meta-Analyst (version 3; BioStat Inc., USA). Dichotomous endpoints (e.g., death) from each included study will be pooled and described as risk ratios and 95% confidence intervals. Continuous endpoints will be pooled using standardized mean differences with inverse variance random effects modeling. If included studies with continuous endpoints use similar methods of measurement — mean differences will be used. 

The Cochrane *I*^2^ statistic will be used to assess statistical heterogeneity of effect sizes. We will interpret *I*^2^ using the following thresholds: 0–40% (low heterogeneity), 30–60% (moderate heterogeneity), 50–90% (substantial heterogeneity), and 75–100% (considerable heterogeneity). If considerable heterogeneity is observed (75–100%), the included studies will be investigated using subgroup and sensitivity analyses. We will correct for the multiple use of control groups using the method described by Vesterinin et al. [22]. If extreme outlier data points are observed during analysis of the primary outcome, a sensitivity analysis will be performed on these outliers. To examine heterogeneity of the primary outcome, planned subgroup analyses will be performed, in accordance with risk-of-bias assessments.

### Sex-based analysis

Importantly, it must be noted that the current best practices for conducting sex-based analyses in preclinical systematic reviews are not well defined. There are numerous important factors that must be considered in the design and conduct of these analyses. Investigating the response to treatment between males and females could presumably be done by comparing the differences to baseline sepsis severity (e.g., comparing septic animals that have received intervention vs septic animals not receiving treatment) after administration of an intervention. For example, if administration of a novel pharmaceutical agent decreased male mortality from 100 to 50% while decreasing female mortality from 100 to 30%, it could be concluded that females exhibited a greater response to treatment.

**Fig. 1:**
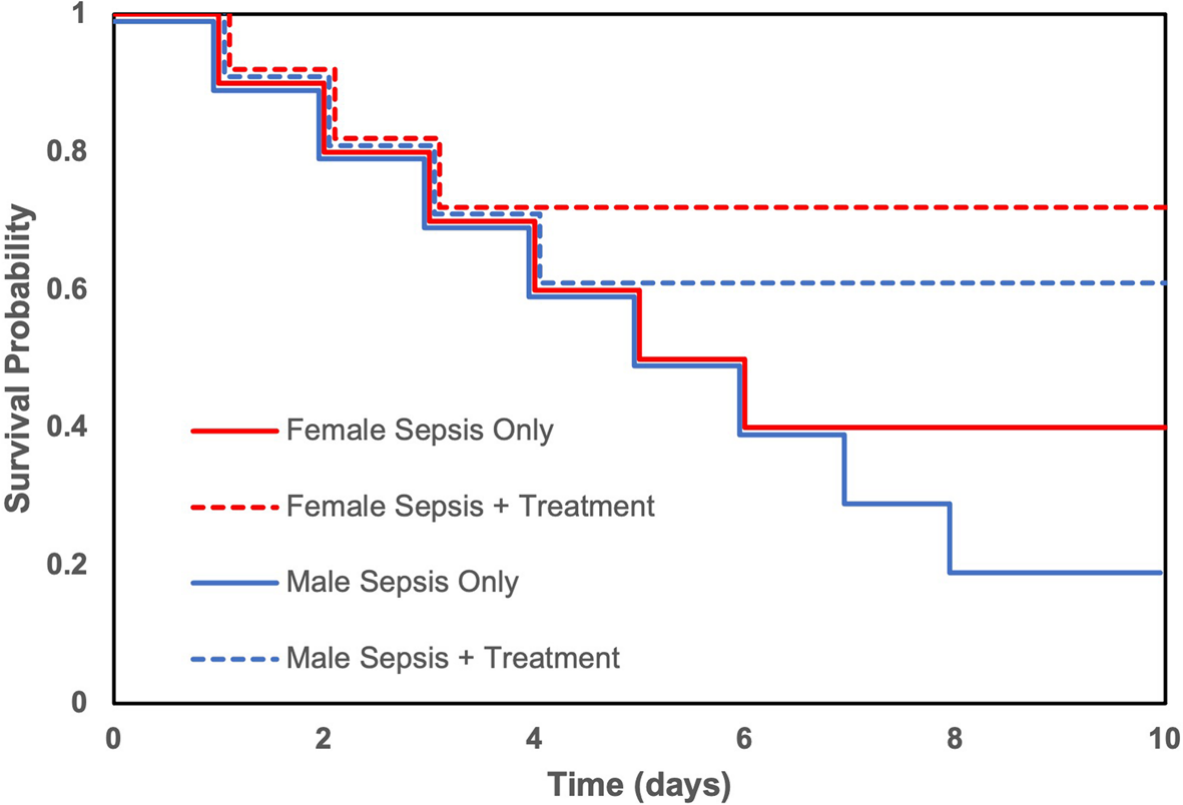
Survival for male and female septic animals receiving an intervention or no intervention in a theoretical example

However, assessing the response to treatment is complicated in practice by baseline differences in the outcome of interest between males and females. For example, consider an intervention that results in a relative 50% decrease in mortality in both males and females (Fig. 1, Table 2). In a sample of 10 male animals with an expected probability of survival at baseline of 20% at the latest recorded timepoint (i.e., 8 animals die), administration of this treatment would translate into 4 fewer deaths. In contrast, the same relative 50% decrease in mortality in a group of 10 female animals with an expected probability of survival at baseline of 40% (6 animals die) at the latest recorded timepoint translates into only 3 fewer deaths. In this sense, although the relative decrease in mortality was equivalent in both males and females, the absolute response to treatment was greater in males (4 fewer deaths post-intervention) compared to females (3 fewer deaths post-intervention). Interestingly, the absolute probability of survival post-intervention provides another perspective, demonstrating that post-treatment more females were alive than males (70% versus 60%, respectively).

**Table 2:** Comparison of mortality between males and females in a theoretical example

Outcome	Male (10 animals total)	Female (10 animals total)
Baseline survival	2/10 (20%)	4/10 (40%)
Baseline mortality	8/10 (80%)	6/10 (60%)
Post-intervention — relative decrease in mortality	50%	50%
Post-intervention — deaths avoided	4	3
Post-intervention — absolute survival	60%	70%

Given that each one of these comparisons provide different interpretations of the same data, we will apply three separate approaches to compare our prespecified outcomes in males and females. Firstly, we will compare male animals at baseline vs female animals at baseline (i.e., sepsis only, with no intervention of interest). Secondly, to assess the relative response to treatment, the magnitude of effect of the intervention (in mean differences) compared to baseline (i.e., animals not receiving intervention) will be calculated for both males and females. The resultant mean difference will be compared between males and females (i.e., difference of mean differences). Finally, we will conduct an absolute comparison of male vs female animals post-intervention (i.e., male animals post-intervention versus female animals post-intervention). Furthermore, primary analyses will be stratified by the age of included animals (e.g., neonatal, adult, or aged). These stratified analyses will allow us to explore the potential effects of sex hormones, which are differentially expressed depending on age.

Specifically in the investigation of sex-dependent differences in sepsis, many individual studies have suggested that females demonstrate better outcomes than males; however, formal meta-analysis has not yet been performed to summarize these findings. Furthermore, these comparisons are complicated by significant variations in important factors that directly affect pathogenesis and outcomes of sepsis, such as the model of sepsis, severity of disease, and administration of foundational therapies (e.g., fluids and antibiotics). These factors must be accounted for in these analyses.

### Subgroup analyses

Several exploratory subgroup analyses are planned. These include the following: sepsis model (i.e., cecal ligation and puncture, fecal peritonitis), host species (i.e., small versus large animal models), and intervention (i.e., conventional/“easily-administered” interventions that could be easily and readily adapted for therapeutic administration to humans versus experimental/ “difficult to administer” interventions that would require considerable development and consideration for use in humans (e.g., products of specific knockout/knock-in models)).

### Meta-biases assessment

Evaluation for publication bias will be conducted using Egger’s regression test and visual inspection of funnel plots, generated by Comprehensive Meta-Analyst (version 3; BioStat Inc., USA).

### Knowledge dissemination

Findings from this proposed systematic review will directly impact the design and focus of a planned multicenter preclinical study by National Preclinical Sepsis Platform (NPSP; funded by Sepsis Canada) to investigate sex-dependent differences in preclinical sepsis [23]. We anticipate the publication of our systematic review in a peer-reviewed, open-access journal at the conclusion of this project. Study results will be presented at national/international conferences and social media and shared with key stakeholders, including the Canadian Critical Care Translational Biology Group [24].

## Discussion

In 2009, it was demonstrated that male sex bias was evident in 8 of 10 biomedical disciplines studied [25]. In a 10-year follow-up, there was no change observed in the proportion of studies that included data analyzed by sex in 8 of 9 disciplines [26]. Factors that may contribute to the underappreciation of sex and gender in biomedical research include, but are not limited to, the misunderstanding of terminology, financial motives, experimental design considerations, and inherent biases. This sex bias in biomedical research is especially concerning given the consistently reported sex-dependent differences in the pathogenesis and outcomes of major disease [27–29], pharmacokinetics and pharmacodynamics [30], and treatment responses [31–33].

Our systematic review will identify and summarize preclinical studies that investigate sex-dependent responses to therapy in animal models of sepsis. This work will expand on our recent systematic review highlighting the paucity of preclinical studies investigating sex-dependent responses to antibiotic and fluid therapy in sepsis [6]. Given that the importance of investigating sex- and gender-dependent differences has recently been emphasized by major funding agencies, we anticipate that a number of relevant studies will be captured in our search. Given the relative lack of guidance surrounding sex-based analyses in preclinical systematic reviews, we hope that our study will contribute towards the development of best practices to inform the design and conduct of future studies. For example, we emphasize here that multiple interpretations of the same data (using different methods of data analysis) are necessary to comprehensively evaluate and communicate sex-dependent differences in response to sepsis treatments.

Our systematic review is informed by an 'integrated knowledge translation' approach [23], where knowledge users will contribute to the research. Specficially, members of Sepsis Canada's NPSP are involved in the design and execution of this systematic review and will contribute to the dissemination of findings. Indeed, findings from this systematic review will directly impact the design and conduct of a planned multicenter preclinical study by the NPSP to investigate sex-dependent differences in preclinical sepsis [23]. Our systematic review will provide a comprehensive assessment of male versus female differences in response to a variety of sepsis therapies and provide insight into “baseline” sex-dependent differences between untreated male and female septic animals, reflecting the natural progression of sepsis. Furthermore, it will also allow for the identification of important knowledge gaps and provide an overview of potential mechanisms that may underlie biological sex-dependent differences.

A foundational understanding of baseline sex-dependent differences in sepsis is necessary prior to the design and conduct of future interventional preclinical and clinical studies. As such, our systematic review will serve as an important early step in advancing our shared goal of improving “bench-to-bedside” translation of sepsis therapies.

## Supplementary Information

**Additional file 1. Appendix 1: **PRISMA-P 2015 Checklist.** Appendix 2: **Sample Search Strategy.

## Data Availability

All the data from this study will be openly available.
