# Supplementary material for: Sex-based analysis of treatment responses in animal models of sepsis: a preclinical systematic review protocol
Source: Syst Rev. 2023 Mar 21;12:50. doi: 10.1186/s13643-023-02189-2 (PMC10029211; doi:10.1186/s13643-023-02189-2)
Supplement: Supplementary file 1 — Additional file 1. Appendix 1: PRISMA-P 2015 Checklist. Appendix 2: Sample Search Strategy. [file 13643_2023_2189_MOESM1_ESM.pdf]

Supplemental Files

Appendix 1: PRISMA-P 2015 Checklist

Section/topic	#	Checklist item	Information reported		Line number(s)
			Yes	No	
**ADMINISTRATIVE INFORMATION**					
**Title**					
Identification	1a	Identify the report as a protocol of a systematic review	☒	☐	2
Update	1b	If the protocol is for an update of a previous systematic review, identify as such	☐	☒	N/A
**Registration**	2	If registered, provide the name of the registry (e.g., PROSPERO) and registration number in the Abstract	☒	☐	PROSPERO Registration Number Pending
**Authors**					
Contact	3a	Provide name, institutional affiliation, and e-mail address of all protocol authors; provide physical mailing address of corresponding author	☒	☐	4-46, 68-75
Contributions	3b	Describe contributions of protocol authors and identify the guarantor of the review	☒	☐	69-76, 98-104
**Amendments**	4	If the protocol represents an amendment of a previously completed or published protocol, identify as such and list changes; otherwise, state plan for documenting important protocol amendments	☐	☒	N/A
**Support**					
Sources	5a	Indicate sources of financial or other support for the review	☒	☐	86-96
Sponsor	5b	Provide name for the review funder and/or sponsor	☒	☐	86-96
Role of sponsor/funder	5c	Describe roles of funder(s), sponsor(s), and/or institution(s), if any, in developing the protocol	☒	☐	86-96
**INTRODUCTION**					
**Rationale**	6	Describe the rationale for the review in the context of what is already known	☒	☐	141-156

Section/topic	#	Checklist item	Information reported		Line number(s)
			Yes	No	
**Objectives**	7	Provide an explicit statement of the question(s) the review will address with reference to participants, interventions, comparators, and outcomes (PICO)	☒	☐	167-176
**METHODS**					
**Eligibility criteria**	8	Specify the study characteristics (e.g., PICO, study design, setting, time frame) and report characteristics (e.g., years considered, language, publication status) to be used as criteria for eligibility for the review	☒	☐	214-224
**Information sources**	9	Describe all intended information sources (e.g., electronic databases, contact with study authors, trial registers, or other grey literature sources) with planned dates of coverage	☒	☐	276-289
**Search strategy**	10	Present draft of search strategy to be used for at least one electronic database, including planned limits, such that it could be repeated	☒	☐	291-301, Appendix 1
STUDY RECORDS					
Data management	11a	Describe the mechanism(s) that will be used to manage records and data throughout the review	☒	☐	304-306
Selection process	11b	State the process that will be used for selecting studies (e.g., two independent reviewers) through each phase of the review (i.e., screening, eligibility, and inclusion in meta-analysis)	☒	☐	308-320
Data collection process	11c	Describe planned method of extracting data from reports (e.g., piloting forms, done independently, in duplicate), any processes for obtaining and confirming data from investigators	☒	☐	322-333
**Data items**	12	List and define all variables for which data will be sought (e.g., PICO items, funding sources), any pre-planned data assumptions and simplifications	☒	☐	322-333
**Outcomes and prioritization**	13	List and define all outcomes for which data will be sought, including prioritization of main and additional outcomes, with rationale	☒	☐	250-274
**Risk of bias in individual studies**	14	Describe anticipated methods for assessing risk of bias of individual studies, including whether this will be done at the outcome or study level, or both; state how this information will be used in data synthesis	☒	☐	334-344
DATA					
**Synthesis**	15a	Describe criteria under which study data will be quantitatively synthesized	☒	☐	346-368

Section/topic	#	Checklist item	Information reported		Line number(s)
			Yes	No	
	15b	If data are appropriate for quantitative synthesis, describe planned summary measures, methods of handling data, and methods of combining data from studies, including any planned exploration of consistency (e.g., I^2, Kendall's tau)	☒	☐	346-433
	15c	Describe any proposed additional analyses (e.g., sensitivity or subgroup analyses, meta-regression)	☒	☐	421-428
	15d	If quantitative synthesis is not appropriate, describe the type of summary planned	☒	☐	355-357
**Meta-bias(es)**	16	Specify any planned assessment of meta-bias(es) (e.g., publication bias across studies, selective reporting within studies)	☒	☐	430-433
**Confidence in cumulative evidence**	17	Describe how the strength of the body of evidence will be assessed (e.g., GRADE)	☐	☒	N/A. GRADE is not applicable to animal studies.

Appendix 2: Sample Search Strategy

Ovid MEDLINE(R) ALL <1946 to August 17, 2021>

1 Drug Evaluation, Preclinical/ 53029
2 exp models, animal/ 601799
3 exp animals, laboratory/ 911889
4 (preclinic* or pre clinic*).tw,kw. 127591
5 (animals or animal or mice or mus or mouse or murine or woodmouse or rats or rat or murinae or muridae or cottonrat or cottonrats or hamster or hamsters or cricetinae or rodentia or rodent or rodents or pigs or pig or swine or swines or piglets or piglet or boar or boars or "sus scrofa" or ferrets or ferret or polecat or polecats or "mustela putorius" or "guinea pigs" or "guinea pig" or cavia or callithrix or marmoset or marmosets or cebuella or hapale or octodon or chinchilla or chinchillas or gerbillinae or gerbil or gerbils or jird or jirds or merione or meriones or rabbits or rabbit or hares or hare or diptera or flies or fly or dipteral or drosphila or drosophilidae or cats or cat or carus or felis or nematoda or nematode or nematoda or nematode or nematodes or sipunculida or dogs or dog or canine or canines or canis or sheep or sheeps or mouflon or mouflons or ovis or goats or goat or capra or capras or rupicapra or chamois or haplorhini or monkey or monkeys or anthropoidea or anthropoids or saguinus or tamarin or tamarins or leontopithecus or hominidae or ape or apes or pan or paniscus or "pan paniscus" or bonobo or bonobos or troglodytes or "pan troglodytes" or gibbon or gibbons or siamang or siamangs or nomascus or symphalangus or chimpanzee or chimpanzees or prosimians or "bush baby" or prosimian or bush babies or galagos or galago or pongidae or gorilla or gorillas or pongo or pygmaeus or "pongo pygmaeus" or orangutans or pygmaeus or lemur or lemurs or lemuridae or horse or horses or pongo or equus or cow or calf or bull or chicken or chickens or gallus or quail or bird or birds or quails or poultry or poultries or fowl or fowls or reptile or reptilia or reptiles or snakes or snake or lizard or lizards or alligator or alligators or crocodile or crocodiles or turtle or turtles or amphibian or amphibians or amphibia or frog or frogs or bombina or salientia or toad or toads or "epidalea calamita" or salamander or salamanders or eel or eels or fish or fishes or pisces or catfish or catfishes or siluriformes or arius or heteropneustes or sheatfish or perch or perches or percidae or perca or trout or trouts or char or chars or salvelinus or "fathead minnow" or minnow or cyprinidae or carps or carp or zebrafish or zebrafishes or goldfish or goldfishes or guppy or guppies or chub or chubs or tinca or barbels or barbus or pimephales or promelas or "poecilia reticulata" or mullet or mullets or seahorse or seahorses or mugil curema or atlantic cod or shark or sharks or catshark or anguilla or salmonid or salmonids or whitefish or whitefishes or salmon or salmons or sole or solea or "sea lamprey" or lamprey or lampreys or pumpkinseed or sunfish or sunfishes or tilapia or tilapias or turbot or turbots or flatfish or flatfishes or sciuridae or squirrel or squirrels or chipmunk or chipmunks or suslik or susliks or vole or voles or lemming or lemmings or muskrat or muskrats or lemmus or otter or otters or marten or martens or martes or weasel or badger or badgers or ermine or mink or minks or sable or sables or gulo or gulos or wolverine or wolverines or minks or mustela or llama or llamas or alpaca or alpacas or camelid or camelids or guanaco or guanacos or chiroptera or chiropteras or bat or bats or fox or foxes or iguana or iguanas or xenopus

laevis or parakeet or parakeets or parrot or parrots or donkey or donkeys or mule or mules or zebra or zebras or shrew or shrews or bison or bisons or buffalo or buffaloes or deer or deers or bear or bears or panda or pandas or "wild hog" or "wild boar" or fitchew or fitch or beaver or beavers or jerboa or jerboas or capybara or capybaras).tw,kf. 5105641
6 exp Animals/ not humans/ 4874719
7 or/1-6 6778779
8 ((sex or gender) adj2 (difference* or disparit*)).tw. 88219
9 sex* dimorph*.tw,kw. 15667
10 (gender difference* or gender disparit*).kw. 3391
11 (sex or gender).ti. 149120
12 (gender based or gender specific).tw,kw. 16261
13 Sex Factors/ 273124
14 Sex Characteristics/ 57300
15 (male* and female*).tw,kw. 511499
16 Female/ and male/ 6114765
17 or/8-16 6305690
18 7 and 17 787483
19 exp Systemic Inflammatory Response Syndrome/ 135633
20 (sepsis or septic* or bacteremia or bacteraemia or endotoxic* or endotoxemi* or endotoxaemi*).tw,kw. 196041
21 cecum/ and ligation/ and punctures/ 447
22 ((cecum or cecal or coecum or caecum) adj3 (ligation* or puncture* or injur*)).tw. 4827
23 ((cecum or cecal or coecum or caecum) and (ligation* or puncture* or injur*)).kf. 246
24 (Peritonitis/ or peritonitis.tw,kw.) and (feces/ or feces.tw,kw. or fecal.tw,kw. or faecal.tw,kw. or faeces.tw,kw.) 1159
25 slurry.tw,kw. 6466
26 fecal stem solution.tw,kw. 3
27 (Injections, Intraperitoneal/ or Infusions, Parenteral/) and (feces/ or feces.tw,kw. or fecal.tw,kw. or faecal.tw,kw. or faeces.tw,kw.) 576
28 ((intraperiton* or periton*) adj10 (fecal or faecal or fece* or faece*)).tw. 1004
29 or/19-28 257578
30 18 and 29 6825
31 (2021012* or 2020013* or 202103* or 202104* or 202105* or 202106* or 202107* or 202108*).dt. 807776
32 30 and 31 41
33 remove duplicates from 32 40

Embase Classic+Embase <1947 to 2021 August 17>

1 exp animal experiment/ or exp animal model/ or exp experimental animal/ or exp transgenic animal/ or exp male animal/ or exp female animal/ or exp juvenile animal/ or animal/ or chordata/ or vertebrate/ or tetrapod/ or exp fish/ or amniote/ or exp amphibia/

or mammal/ or exp reptile/ or exp sauropsid/ or therian/ or exp monotremate/ or placental mammals/ or exp marsupial/ or Euarchontoglires/ or exp Afrotheria/ or exp Boreoeutheria/ or exp Laurasiatheria/ or exp Xenarthra/ or primate/ or exp Dermoptera/ or exp Glires/ or exp Scandentia/ or Haplorhini/ or exp prosimian/ or simian/ or exp tarsiiform/ or Catarrhini/ or exp Platyrrhini/ or ape/ or exp Cercopithecidae/ or hominid/ or exp hylobatidae/ or exp chimpanzee/ or exp gorilla/ or exp orang utan/ 7121435

2 (animal or animals or pisces or fish or fishes or catfish or catfishes or sheatfish or silurus or arius or heteropneustes or clarias or gariepinus or fathead minnow or fathead minnows or pimephales or promelas or cichlidae or trout or trouts or char or chars or salvelinus or salmo or oncorhynchus or guppy or guppies or millionfish or poecilia or goldfish or goldfishes or carassius or auratus or mullet or mullets or mugil or curema or shark or sharks or cod or cods or gadus or morhua or carp or carps or cyprinus or carpio or killifish or eel or eels or anguilla or zander or sander or lucioperca or stizostedion or turbot or turbots or psetta or flatfish or flatfishes or plaice or pleuronectes or platessa or tilapia or tilapias or oreochromis or sarotherodon or common sole or dover sole or solea or zebrafish or zebrafishes or danio or rerio or seabass or dicentrarchus or labrax or morone or lamprey or lampreys or petromyzon or pumpkinseed or pumpkinseeds or lepomis or gibbosus or herring or clupea or harengus or amphibia or amphibian or amphibians or anura or salientia or frog or frogs or rana or toad or toads or bufo or xenopus or laevis or bombina or epidalea or calamita or salamander or salamanders or newt or newts or triturus or reptilia or reptile or reptiles or bearded dragon or pogona or vitticeps or iguana or iguanas or lizard or lizards or anguis fragilis or turtle or turtles or snakes or snake or aves or bird or birds or quail or quails or coturnix or bobwhite or colinus or virginianus or poultry or poultries or fowl or fowls or chicken or chickens or gallus or zebra finch or taeniopygia or guttata or canary).tw.
2153250

3 (canaries or serinus or canaria or parakeet or parakeets or grasskeet or parrot or parrots or psittacine or psittacines or shelduck or tadorna or goose or geese or branta or leucopsis or woodlark or lullula or flycatcher or ficedula or hypoleuca or dove or doves or geopelia or cuneata or duck or ducks or greylag or graylag or anser or harrier or circus pygargus or red knot or great knot or calidris or canutus or godwit or limosa or lapponica or meleagris or gallopavo or jackdaw or corvus or monedula or ruff or philomachus or pugnax or lapwing or peewit or plover or vanellus or swan or cygnus or columbianus or bewickii or gull or chroicocephalus or ridibundus or albifrons or great tit or parus or aythya or fuligula or streptopelia or risoria or spoonbill or platalea or leucorodia or blackbird or turdus or merula or blue tit or cyanistes or pigeon or pigeons or columba or pintail or anas or starling or sturnus or owl or athene noctua or pochard or ferina or cockatiel or nymphicus or hollandicus or skylark or alauda or tern or sterna or teal or crecca or oystercatcher or haematopus or ostralegus or shrew or shrews or sorex or araneus or crocidura or russula or european mole or talpa or chiroptera or bat or bats or eptesicus or serotinus or myotis or dasycneme or daubentonii).tw.
101237

4 (pipistrelle or pipistrellus or cat or cats or felis or catus or feline or dog or dogs or canis or canine or canines or otter or otters or lutra or badger or badgers or meles or fitchew or fitch or foumart or foulmart or ferrets or ferret or polecat or polecats or mustela or putorius or weasel or weasels or fox or foxes or vulpes or common seal or

phoca or vitulina or grey seal or halichoerus or horse or horses or equus or equine or equidae or donkey or donkeys or mule or mules or pig or pigs or swine or swines or hog or hogs or boar or boars or porcine or piglet or piglets or sus or scrofa or llama or llamas or lama or glama or deer or deers or cervus or elaphus or cow or cows or bos taurus or bos indicus or bovine or bull or bulls or cattle or bison or bisons or sheep or sheeps or ovis aries or ovine or lamb or lambs or mouflon or mouflons or goat or goats or capra or caprine or chamois or rupicapra or leporidae or lagomorpha or lagomorph or rabbit or rabbits or oryctolagus).tw. 1800815

5 (cuniculus or laprine or hares or lepus or rodentia or rodent or rodents or murinae or mouse or mice or mus or musculus or murine or woodmouse or apodemus or rat or rats or rattus or norvegicus or guinea pig or guinea pigs or cavia or porcellus or hamster or hamsters or mesocricetus or cricetulus or cricetus or gerbil or gerbils or jird or jirds or meriones or unguiculatus or jerboa or jerboas or jaculus or chinchilla or chinchillas or beaver or beavers or castor fiber or castor canadensis or sciuridae or squirrel or squirrels or sciurus or chipmunk or chipmunks or marmot or marmots or marmota or suslik or susliks or spermophilus or cynomys or cottonrat or cottonrats or sigmodon or vole or voles or microtus or myodes or glareolus or primate or primates or prosimian or prosimians or lemur or lemurs or lemuridae or loris or bush baby or bush babies or bushbaby or bushbabies or galago or galagos or anthropoidea or anthropoids or simian or simians).tw. 3898572

6 (monkey or monkeys or marmoset or marmosets or callithrix or cebuella or tamarin or tamarins or saguinus or leontopithecus or squirrel monkey or squirrel monkeys or saimiri or night monkey or night monkeys or owl monkey or owl monkeys or douroucoulis or aotus or spider monkey or spider monkeys or ateles or baboon or baboons or papio or rhesus monkey or macaque or macaca or mulatta or cynomolgus or fascicularis or green monkey or green monkeys or chlorocebus or vervet or vervets or pygerythrus or hominoidea or ape or apes or hylobatidae or gibbon or gibbons or siamang or siamangs or nomascus or symphalangus or hominidae or orangutan or orangutans or pongo or chimpanzee or chimpanzees or pan troglodytes or bonobo or bonobos or pan paniscus or gorilla or gorillas or troglodytes).tw. 171257

7 (preclinic* or pre clinic*).tw. 194265

8 1 or 2 or 3 or 4 or 5 or 6 or 7 8479657

9 sex difference/ or sex factor/ 422792

10 ((sex or gender) adj2 (difference* or disparit*)).tw. 119889

11 sex* dimorph*.tw. 17432

12 (sex or gender).ti. 182956

13 (gender based or gender specific).tw. 21817

14 sexual characteristics/ 2283

15 (male* and female*).tw. 807578

16 (exp female animal/ or female/) and (exp male animal/ or male/) 7167862

17 or/9-16 7418356

18 8 and 17 864432

19 exp systemic inflammatory response syndrome/ 309153

20 (sepsis or septic* or bacteremia or bacteraemia or endotoxic* or endotoxemi* or endotoxaemi*).tw. 288551

21 systemic inflammat*.tw. 54229

22 (peritonitis and (feces or fecal or faecal or faeces)).tw. 1586
23 ((intraperiton* or periton*) adj10 (fecal or faecal or fece* or faece*)).tw. 1541
24 slurry.tw. 8481
25 fecal stem solution.tw. 5
26 peritonitis/ and feces/ 462
27 experimental sepsis/ or "cecal ligation and puncture-induced sepsis"/ or experimental septic shock/ 2326
28 or/19-27 448452
29 18 and 28 9537
30 conference abstract.pt. 4155164
31 conference review.pt. 13244
32 29 not (30 or 31) 6970
33 limit 32 to yr="2017 -Current" 2216
34 (2021012* or 2020013* or 202103* or 202104* or 202105* or 202106* or 202107* or 202108*).dc. 1328278
35 33 and 34 485
